# A Young Man With Apical Fibrotic Changes on Chest Imaging and History of Pneumothorax

**DOI:** 10.1016/j.chpulm.2026.100248

**Published:** 2026-03-06

**Authors:** Ilias E. Dimeas, Paraskevi Kirgou, Konstantina Papacharalampous, Cormac McCarthy, Zoe Daniil

**Affiliations:** a School of Medicine, University College Dublin, Dublin, Ireland; b Department of Respiratory Medicine, St. Vincent’s University Hospital, Dublin, Ireland; c Department of Respiratory Medicine, Faculty of Medicine, University of Thessaly, Biopolis, Larissa, Greece; d Pathology Department, IASO Thessalias General Hospital, Larissa, Greece

## Abstract

A 37-year-old man, who actively smoked with a 15 pack-year history, working in a greenhouse, was referred for evaluation of intermittent, nonpleuritic chest pain without specific characteristics. He reported no dyspnea, weight loss, hemoptysis, or fever. His medical history was unremarkable except for a left spontaneous pneumothorax 3 years earlier that failed to resolve with chest-tube drainage and required surgical bullectomy with apical and pleural biopsy, for which no diagnosis was established despite thorough evaluation. A chest radiograph during the patient’s first hospitalization demonstrated a large, left-sided pneumothorax with partial lung collapse (Fig 1A), for which a chest tube was inserted; however, the lung could not be fully reexpanded (Fig 1B). A subsequent chest high-resolution CT scan (Fig 2) revealed bilateral apical pleural thickening and persistent left pneumothorax with subpleural parenchymal collapse. Because of the incomplete expansion, the patient underwent left apical bullectomy and pleurectomy through a limited thoracotomy. He recovered uneventfully and was discharged after a few days. Surgical specimens included an apical cap and visceral pleura. Histologic examination revealed dense pleural and subpleural fibrosis with an abrupt transition to relatively preserved underlying alveolar parenchyma (Fig 3). These findings were interpreted as nonspecific chronic pleuritis, and a definitive diagnosis could not be established at that time. He was not taking any medications, and no occupational exposure was mentioned. There was no family history of interstitial lung disease. The patient was referred to our hospital to investigate and manage his long-standing condition.

## Physical Examination Findings

At presentation, the patient appeared mildly slender (BMI, 18.7 kg/m^2^) with slight platythorax but no digital clubbing. A well-healed thoracotomy scar was noted over the left hemithorax. His vital signs were stable, and oxygen saturation was 98% on room air. Chest, face, and skin inspection showed no deformity or rash, and breath sounds were clear and symmetrical bilaterally without crackles or wheezes. There were no features of connective tissue disease such as sclerodactyly, joint swelling, or telangiectasia. No pathologic heart sounds were detected, and no other abnormalities were found.

## Diagnostic Studies

On referral to our center, 3 years after initial pneumothorax, for recurrent chest discomfort, a repeat chest radiograph was normal (Fig 4). However, chest HRCT scan (Fig 5) demonstrated bilateral apical pleural thickening with adjacent mild subpleural reticulation and subtle tractional bronchiectasis, more pronounced on the left side. There was no honeycombing, ground-glass opacity, or mediastinal lymphadenopathy. Pulmonary function testing demonstrated normal spirometry and near-normal lung volumes (FVC, 90.5% predicted; FEV_1_, 92.0% predicted; FEV_1_/FVC ratio, 0.85; total lung capacity [TLC], 117% predicted), with a markedly elevated residual volume (RV) to TLC ratio (173% predicted) and moderately reduced diffusing capacity for carbon monoxide (Dlco) (55% predicted). Arterial blood gas analysis demonstrated normal oxygenation at rest and on exertion.

**Figure 1:**
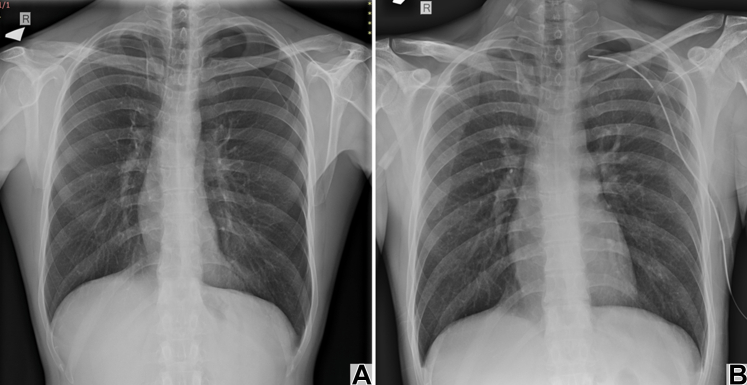
A, B, Chest radiographs from initial presentation 3 y earlier: (A) large, left-sided pneumothorax with partial lung collapse during the patient’s hospitalization, and (B) chest tube in place, with incomplete reexpansion of the left lung.

**Figure 2:**
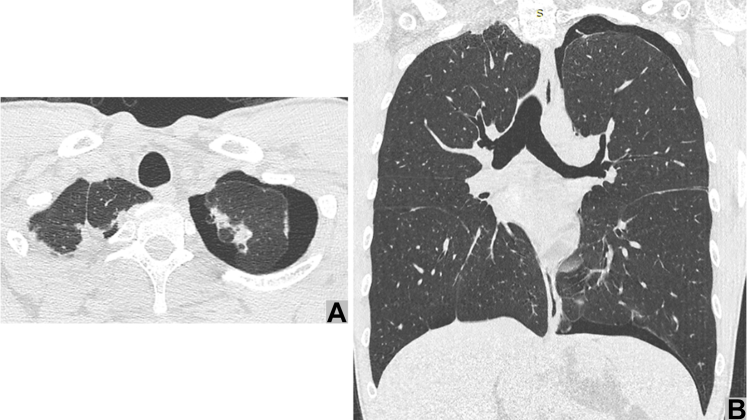
A, B, Chest high-resolution CT scan from initial presentation 3 y earlier: (A) axial and (B) coronal reconstructions showing bilateral apical pleural thickening with a persistent left-sided pneumothorax.

**Figure 3:**
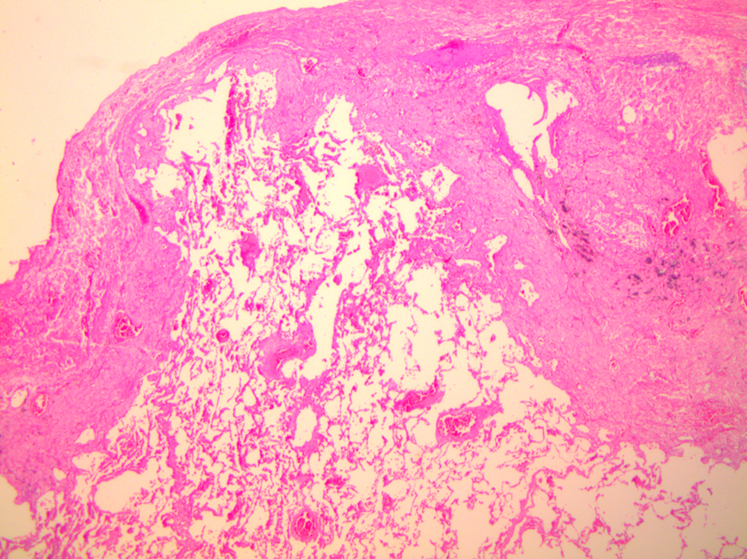
Histologic section from the left lung apical cap and visceral pleura showing dense pleural and subpleural fibrosis with an abrupt transition to relatively preserved underlying alveolar parenchyma (hematoxylin-eosin, original magnification ×2.5).

**Figure 4:**
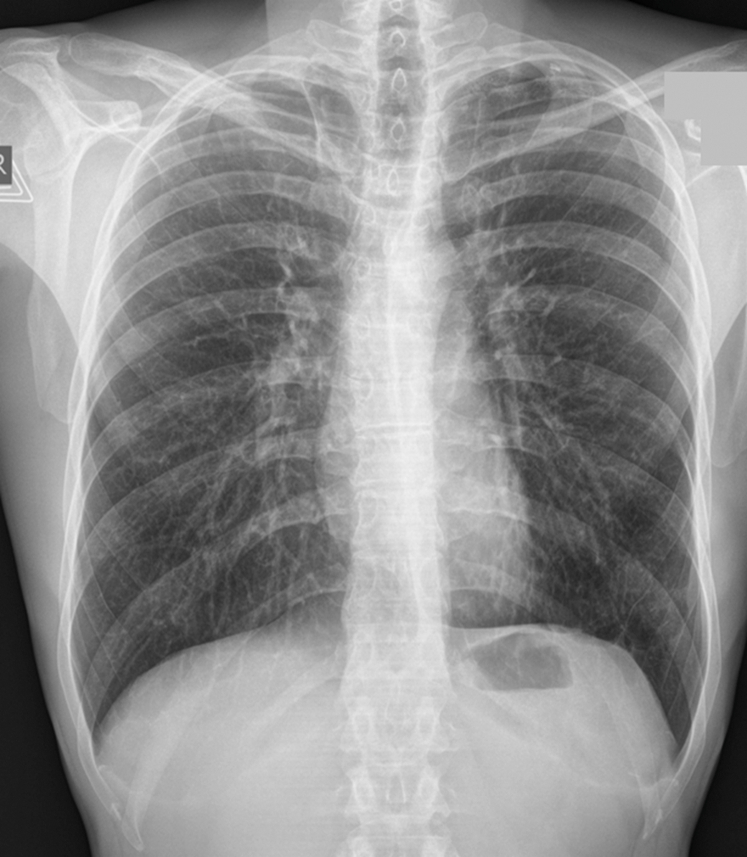
Follow-up chest radiograph on referral to our center. Complete resolution of the prior pneumothorax, with full reexpansion of the left lung.

**Figure 5:**
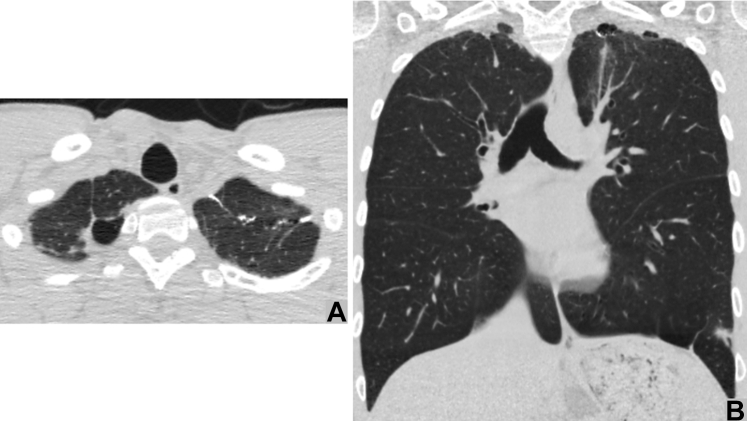
A, B, Chest high-resolution CT scan on referral to our center: (A) axial and (B) coronal reconstructions showing bilateral apical pleural thickening with adjacent mild subpleural reticulation and subtle tractional bronchiectasis, more pronounced on the left side.

Given the longitudinal clinical course, several possibilities were considered; therefore, a comprehensive evaluation was performed. There was no history of transplantation, thoracic radiotherapy, cytotoxic chemotherapy, ARDS, or severe pneumonia, and no occupational exposure to asbestos, silica, talc, or metallic dusts. Serologic studies for connective tissue disease, *Aspergillus* serology, and hypersensitivity pneumonitis precipitins were negative, and bronchoscopic cultures for mycobacteria and fungi were also negative. Calprotectin testing and the absence of gastrointestinal symptoms excluded inflammatory bowel disease, and telomere analysis showed no abnormality.

**Figure 6:**
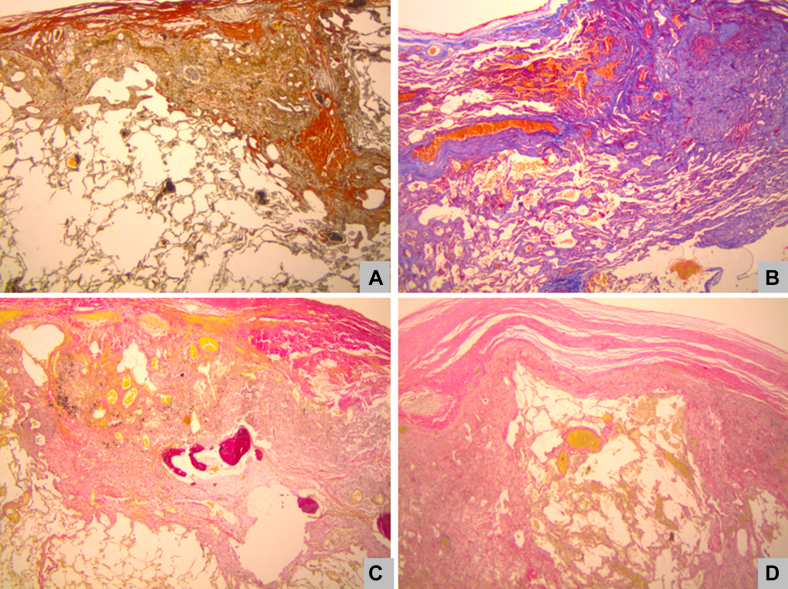
A-D, Histologic sections from the left lung apical cap and visceral pleura stained with special histochemical techniques: (A) pleural and subpleural fibrosis highlighted by reticulin stain; (B) visceral pleural, intraalveolar, and intimal fibrosis demonstrated by Masson trichrome stain; and (C, D) pleural and subpleural fibrosis with prominent alveolar-septal elastosis highlighted by Verhoeff-Van Gieson stain. (A, B: original magnification ×2.5; C, D: original magnification ×4)

The previous surgical biopsy, which had shown dense subpleural fibrosis with focal involvement of the underlying alveolar structures, had been considered nonspecific, but considering the imaging and physiological pattern, it warranted further review. Considering the unfavorable risk-benefit ratio of performing a new biopsy, the archived surgical specimen was retrieved and underwent additional review with special histochemical stains, including reticulin, Masson trichrome, and Verhoeff-Van Gieson elastic stains, revealing dense pleural fibrosis with collapsed and collagen-filled alveoli and prominent alveolar-septal elastosis (Fig 6), with deeper structural involvement. These findings established the final histopathologic diagnosis.

*What is the diagnosis?*

*Diagnosis:* Idiopathic pleuroparenchymal fibroelastosis

## Discussion

Pleuroparenchymal fibroelastosis is a distinct form of fibrosing interstitial pneumonia characterized by dense subpleural fibroelastosis that predominantly affects the upper lobes. Radiologically, the disease presents with pleural thickening and architectural distortion, progressing to upper lobe volume loss, elevation of the hila, and flattening of the chest wall. Traction bronchiectasis, fissural involvement, and upward shift of the interlobar fissures are frequent. Histologically, pleuroparenchymal fibroelastosis (PPFE) shows dense collagen deposition and marked elastosis of the alveolar septa extending from the visceral pleura into the underlying parenchyma. The interface between affected and normal lung is usually abrupt, and lower lobes are relatively spared or show a secondary pattern such as usual interstitial pneumonia. Clinically, patients are typically slender, with a low BMI and a flattened thoracic cage, and most have never smoked. Progressive exertional dyspnea, dry cough, and recurrent spontaneous pneumothorax are common.

The cornerstone for diagnosis is a combination of high-resolution CT (HRCT) scan and multidisciplinary discussion. HRCT scan demonstrates upper lobe-predominant pleural and subpleural fibrosis with sharp demarcation and tractional distortion, findings that are sufficiently characteristic to allow a confident diagnosis in many cases without the need for surgical biopsy. Histopathologic confirmation, although useful for atypical or unilateral presentations, is often unnecessary because of the high risk of pneumothorax or persistent air leak after biopsy. When tissue is obtained, elastic staining highlights intraalveolar fibroelastosis and visceral pleural fibrosis: the diagnostic gold standard. When tissue sampling is required, lower-risk methods such as cryobiopsy are preferred, and nonessential invasive procedures should be avoided because of the pneumothorax risk. Integration of clinical, radiologic, and pathologic data through multidisciplinary discussion remains the reference approach.

Determining idiopathic vs secondary PPFE is essential because similar radiologic and pathologic changes can occur after lung or bone marrow transplantation, chemotherapy, radiotherapy, chronic infection, inflammatory bowel disease, or autoimmune disease. Long-term imaging follow-up, serologic testing for connective tissue disease, and careful review of treatment and exposure history are required to exclude secondary forms. The distribution pattern may also provide a clue: idiopathic PPFE is usually bilateral and symmetrical, whereas unilateral or localized upper lobe disease strongly suggests a postsurgical or radiation-related process. Screening for prior thoracic surgery, prior radiation, or telomere-biology disorders, such as early graying, cytopenias, or familial pulmonary fibrosis, should be routine in the diagnostic workup. PPFE should also be distinguished from an apical cap or static triangular pleural scar, which typically lack progressive fibroelastotic remodeling and associated physiological abnormalities, and from chronic hypersensitivity pneumonitis or usual interstitial pneumonia overlap, which may coexist. Notably, overlap between idiopathic and secondary phenotypes exists, and recent series highlight a continuum rather than strictly discrete entities.

From a physiological standpoint, PPFE produces a restrictive ventilatory defect with a disproportionately elevated RV/TLC ratio, reflecting compensatory hyperinflation of the lower lobes despite apical volume loss. This feature distinguishes PPFE from other fibrosing interstitial pneumonias and can aid diagnosis even when imaging is subtle. Air trapping within the preserved lung may create shearing forces at the pleural interface, predisposing to pneumothorax, a major complication that can herald disease acceleration or acute decline. Pneumothorax may occur spontaneously or after minor interventions such as bronchoscopy or biopsy and is often recurrent or bilateral. Awareness of this risk is crucial when planning diagnostic or therapeutic procedures. Serial pulmonary function testing every 3 to 6 months, with explicit tracking of RV/TLC in addition to FVC and Dlco, is recommended; a rising RV/TLC should prompt heightened vigilance for pneumothorax or functional deterioration.

Therapeutic options remain limited because no treatment has been proven to halt disease progression. Corticosteroids and immunosuppressants generally lack efficacy, and antifibrotic agents such as pirfenidone and nintedanib have shown variable benefit in small series. Supportive management is therefore central: smoking cessation, optimization of nutrition, vaccination, pulmonary rehabilitation, and supplemental oxygen, when indicated. Because of the frequent association with cachexia, monitoring of body weight and muscle mass is recommended. Enrollment in structured pulmonary rehabilitation programs and implementation of individualized nutritional plans are encouraged. Antifibrotic therapy may be considered case-by-case in progressive disease despite limited evidence, with documentation of goals and trajectory. Lung transplantation is the only curative option for advanced disease; however, dense apical pleural fibrosis can make explant surgery technically challenging. Early referral for transplant assessment should be considered, particularly when FVC falls < 50% predicted or progressive functional decline is documented.

Long-term follow-up is essential, given the heterogeneous clinical course. Serial spirometry and HRCT scan allow assessment of both progression and complications. Quantitative imaging of upper lobe volume and longitudinal tracking of RV/TLC provide objective markers of deterioration. The anteroposterior to transverse chest-diameter ratio, known as the platythorax index, can serve as an adjunct quantitative marker of progression when reproducible. Median survival ranges from 3 to 8 years, but a subset of patients shows indolent stability for a decade or more. Poor prognostic factors include very low BMI, hypercapnia, coexistent lower lobe fibrosis, and recurrent pneumothorax.

In summary, idiopathic PPFE represents an uncommon but increasingly recognized cause of progressive upper lobe fibrosis; therefore, recognition of its characteristic imaging pattern, integration of clinical and physiological clues, exclusion of secondary triggers, and vigilant monitoring are central to diagnosis and management. Awareness of its complications, particularly pneumothorax, nutritional decline, and progressive platythorax, is vital for ongoing care and follow-up.

### Clinical Course

**Figure 7:**
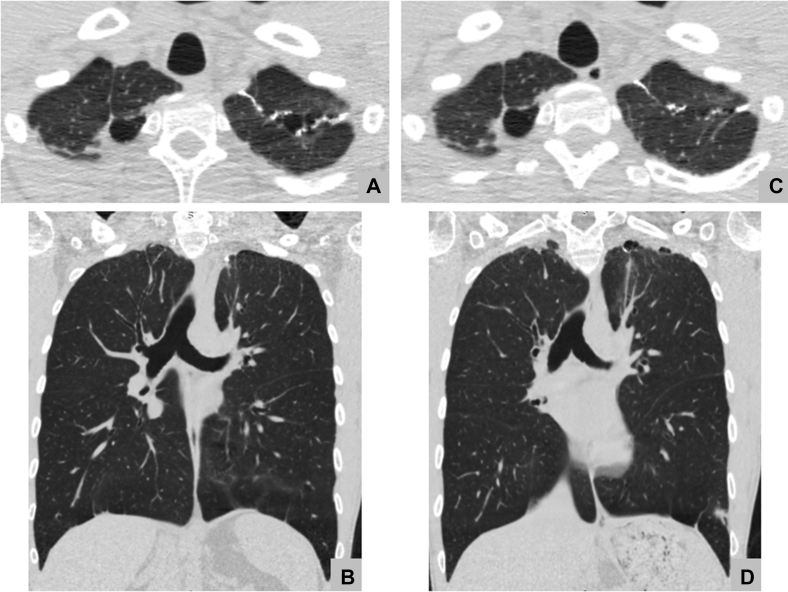
A-D, Chest high-resolution CT scan at 1-year follow-up in our center: (A) axial and (B) coronal reconstructions at follow-up showing stable apical pleural thickening and unchanged pleural and subpleural findings, and (C) axial and (D) coronal reconstructions obtained 1 y earlier, included for comparison.

In this patient, the diagnosis of idiopathic PPFE is supported by the convergence of several defining features: a history of refractory spontaneous pneumothorax, persistent upper lobe-predominant pleural and subpleural fibrosis with sharp demarcation, a characteristic physiological profile with preserved spirometry and disproportionately elevated RV/TLC, histopathologic confirmation of pleural and intraalveolar fibroelastosis on elastic staining, and the absence of clinical, serologic, or exposure-related evidence of secondary causes. Given the pulmonary function profile showing normal spirometry with lung hyperinflation, air trapping and moderately reduced diffusing capacity and the minimal radiologic and symptomatologic change over 3 years, the patient was offered smoking cessation counseling and routine adult vaccinations, both of which he completed. Follow-up pulmonary function testing remained stable 1 year later, with FEV_1_ increasing from 92% to 99% predicted, FVC increasing from 90% to 93% predicted, TLC increasing from 117% to 92% predicted, and Dlco increasing from 55% to 61% predicted, whereas the RV/TLC ratio decreased from 173% to 130%, reducing the risk of recurrent pneumothorax. Follow-up chest HRCT scan performed during the same period showed stable apical pleural thickening compared with the previous scan (Fig 7) and a relatively stable platythorax index from 0.58 to 0.56. The reduction in RV/TLC was interpreted as reflecting dynamic changes in compensatory lower lobe hyperinflation and smoking cessation rather than reversal of pleuroparenchymal fibroelastosis, as radiologic findings remained unchanged. His intermittent, nonpleuritic chest pain had already resolved and was considered incidental. The patient continues to be monitored at regular intervals for potential disease progression or secondary complications.

## Clinical Pearls

1. *Recognize the pattern: upper lobe-predominant pleural fibrosis with architectural distortion and relative sparing of the lower lobes should raise suspicion for PPFE, particularly in patients with prior pneumothorax or persistent apical scarring.*
2. *Interpret physiological findings carefully: an elevated RV/TLC ratio with otherwise preserved spirometry may represent an early physiological signature of PPFE rather than normal lung mechanics.*
3. *Exclude secondary causes: before diagnosing idiopathic PPFE, systematically rule out potential secondary etiologies such as prior transplantation, chemotherapy, radiotherapy, chronic infection, or autoimmune disease.*
4. *Diagnose and monitor wisely: histologic confirmation with elastic stains remains the diagnostic gold standard, but biopsy should be reserved for selected cases because of its high procedural risk. Long-term follow-up with HRCT scan and serial* *pulmonary* *function* *tests*, *particularly tracking RV/TLC and platythorax index, is essential to evaluate disease stability and detect complications.*

## Financial/Nonfinancial Disclosures

None declared.
